# A Single Bout of Intermittent Hypoxia Increases Cerebral Blood Flow and Supports an Executive Function Benefit

**DOI:** 10.1111/psyp.70161

**Published:** 2025-10-07

**Authors:** Denait Haile, Nasimi A. Guluzade, Antonio Mendes, Daniel A. Keir, Matthew Heath

**Affiliations:** ^1^ School of Kinesiology University of Western Ontario London Ontario Canada; ^2^ Graduate Program in Neuroscience University of Western Ontario London Ontario Canada; ^3^ Canadian Centre for Activity and Aging University of Western Ontario London Ontario Canada

**Keywords:** cognition, cortical hemodynamics, inhibitory control, oculomotor, transcranial Doppler ultrasound

## Abstract

Alternating between brief normoxic and hypoxic intervals (i.e., intermittent hypoxia: IH) increases cerebrovascular dilation, cerebral blood flow (CBF), and O_2_ extraction. Some work has shown that the physiological adaptations arising from multiple IH sessions improve brain health and executive function (EF)—a finding linked to a post‐intervention improvement in cortical hemodynamics. Here, we provide a first demonstration of whether the physiological changes associated with a *single* IH session provide a transient post‐intervention EF benefit. Healthy young adults (*N* = 24) completed an IH protocol entailing 12 alternating 5‐min normoxic (P_ET_O_2_ = 100 mmHg) and hypoxic (P_ET_O_2_ = 50 mmHg) intervals that were normocapnic and isocapnic, and on a separate day completed a time‐matched normoxic control protocol. Prior to (T0), and immediately (T1) and 30 min (T2) following each protocol, EF was assessed via the antisaccade task. Antisaccades require a goal‐directed eye movement (i.e., saccade) mirror‐symmetrical to a target and provide the resolution to detect subtle EF changes. As expected, hypoxic intervals decreased arterial and cerebral tissue O_2_ saturation and increased CBF as estimated via near‐infrared spectroscopy and transcranial Doppler ultrasound (*p*s < 0.001). In turn, antisaccade reaction times (RT) did not differ between T0 and T1 (*p* = 0.29); however, at T2 a reliable RT reduction was observed (*p* = 0.004). Notably, cortical hemodynamic changes during the hypoxic intervals did not correlate with the antisaccade RT benefit observed at T2 (*p*s > 0.17). Thus, a single bout of IH provided a transient post‐intervention EF “boost” that was not linked to a unitary physiological adaptation to a reduced O_2_ environment.

## Abstract 2

In the first study to examine the impact of a single session of 5 min alternating intervals of breathing normoxic and hypoxic gas mixtures (i.e., intermittent hypoxia), we found evidence of increased cerebral blood and a post‐protocol improvement in executive function. These findings demonstrate that intermittent hypoxia may serve as a potent framework to support brain health and executive function.

## 1 Introduction

Executive function (EF) includes the core components of inhibitory control, working memory, and cognitive flexibility and is a cognitive construct supported via an extensive frontoparietal network (Diamond 2013; Miyake et al. 2000). An important line of inquiry is identifying the physiological state, or states, benefitting EF given its role in supporting educational and occupational activities. A wealth of evidence has shown that a single bout of exercise and chronic exercise (> 3 months) provide transient (~60‐min) and long‐term EF benefits, respectively (for reviews see Chang et al. 2012; Kramer and Colcombe 2018). In both cases, the benefit has been—in part—linked to an exercise‐based increase in cerebral blood flow (CBF) (Kleinloog et al. 2019; Tari et al. 2020). Indeed, during a single bout of exercise, an exercise‐based increase in CBF has been proposed to elicit thermo‐mechanical changes that enhance processing at the level of local neural circuits (i.e., the hemo‐neural hypothesis) (Moore and Cao 2008), whereas chronic exercise is thought to yield a persistent increase in CBF supporting long‐term structural, mechanical, and biomolecule level changes that enhance EF (Lucas et al. 2012).

Intermittent hypoxia (IH) entails alternating between intervals (typically 2–6 min in duration) of breathing normoxic (i.e., room air) and hypoxic (i.e., FiO_2_ of 10%–13%) gas mixtures and is a protocol that increases CBF and has been identified as a potential intervention to improve brain health (Panza et al. 2023). The onset of a hypoxia interval elicits an acute response wherein a rapid chemoreceptor‐identified reduction in arterial (SaO_2_) and cerebral tissue (ScO_2_) O_2_ saturation stimulates increased ventilation and heart rate (HR) to maintain homeostatic O_2_ delivery (for reviews see Powell et al. 1998; Williams et al. 2022). The acute response leads to dilation of the cerebral arteries (and pial vessels) and decreases cerebrovascular resistance to increase CBF and enhance cerebral O_2_ extraction (Liu et al. 2017; Manukhina et al. 2016; for review see Hoiland et al. 2016).

To the best of our knowledge, only two studies examined whether CBF‐based changes associated with IH impact EF. On the one hand, Zhang et al. (2025) had healthy adults (18–45 years) complete four 10‐min intervals of breathing a hypoxic gas mixture (FiO_2_ = 13%) interspersed with 5‐min of normoxic air for two sessions per day across five successive days (i.e., IH training protocol). Prior to and following the week‐long training protocol, transcranial Doppler ultrasound (TCD) recorded peak middle cerebral artery velocity (MCAv) to estimate CBF, and participants completed three EF tests (i.e., digit span test, Stroop Color, Trail Making Test). Results showed that MCAv increased by ~9% following IH training and was associated with an increased cerebrovascular conductance index (CVCi); however, there were no observed changes in EF. Based on these findings, the authors concluded that IH training improves CBF without impairing EF. On the other hand, Wang et al. (2020) had participants with amnestic mild cognitive impairment (aMCI) complete an IH training protocol involving eight alternating 5‐min hypoxic (FiO_2_ = 10%) and normoxic intervals three times per week for 2 months. The authors recorded mean MCAv and ScO_2_ at baseline and one and two days following completion of the protocol, with a general evaluation of cognition completed at the same time points (i.e., Mini Mental State Exam, digit span test). Although results did not yield a post‐intervention change in MCAv, a 3% post‐intervention increase in ScO_2_ was associated with improved cognitive performance. The authors concluded that IH training improves cerebrovascular O_2_ saturation and extraction and supports improved cognition in a subclinical population (i.e., individuals with aMCI).

In the present work, we examined whether a *single* bout of IH provides an immediate and/or delayed EF benefit. The basis for examining this issue is that the 8–14 cm/s increase in MCAv associated with brief hypoxic intervals (Liu et al. 2017; Zhang et al. 2025; Wang et al. 2020) is on par with the increase observed during very light‐intensity exercise and is a change associated with an EF benefit (Shirzad et al. 2022; Tari et al. 2023; for review see Zou et al. 2023). Thus, if an IH‐based increase in CBF renders mechanical‐ and temperature‐based changes to the neural circuits supporting EF, then it may support a post‐intervention EF benefit comparable to a single bout of exercise. Notably, the present work differs from previous studies examining the impact of IH on EF (i.e., Zhang et al. 2025; Wang et al. 2020) in two important respects. First, we measured arterial CO_2_ tension at a baseline value (i.e., normocapnic) and then used that value to match CO_2_ tension across hypoxic and normoxic intervals (i.e., isocapnic). This is an important control because the hypoxic drive to breathe produces a concomitant decrease in arterial CO_2_ and a hypocapnic‐based cerebrovascular vasoconstriction that decreases CBF (Steinback and Poulin 2008). As such, Zhang et al.'s and Wang et al.'s work—which did not match for between‐interval CO_2_ tensions (i.e., poikilocapnic protocol)—did not provide a paradigm to disentangle how hypoxia and an associated hypocapnic response influence cortical hemodynamics and EF. In particular, the vasoconstriction associated with a poikilocapnic protocol may have blunted the magnitude of an IH‐based increase in CBF and the putative magnitude and unitary expression of an EF benefit. Second, from Zhang et al. and Wang et al., it was concluded that multiple IH sessions are required to elicit an EF benefit; however, it is not known whether a single bout of IH provides an EF benefit. This is a salient issue to address to determine whether the impact of an IH protocol on EF reflects a cumulative and adaptive process or relates to the physiological changes associated with the most recent bout of IH (i.e., a single bout benefit). Accordingly, healthy young adults completed a single bout iso‐ and normocapnic IH protocol involving 12 alternating 5‐min normoxic (P_ET_O_2_ = 100 mmHg) and hypoxic (P_ET_O_2_ = 50 mmHg or ~FiO_2_ = 10%) intervals, and on a separate day an equivalent duration normoxic protocol (i.e., control) was completed. Respiratory, cardiovascular, and cortical hemodynamic (TCD and near‐infrared spectroscopy: NIRS) measures were recorded during IH and control protocols, and for each protocol, EF was evaluated prior to (T0), and immediately (T1) and 30‐min (T2) after protocol cessation. Antisaccades were used to assess EF and are a task requiring a goal‐directed eye movement (i.e., saccade) mirror‐symmetrical to an exogenously presented target. The basis for using the antisaccade task was twofold. First, antisaccades are mediated via frontal EF networks (for reviews see Munoz and Everling 2004) that show task‐dependent changes following a single bout of exercise (for review see Herold et al. 2020). Second, antisaccades provide the resolution to detect subtle EF changes not identified by other EF metrics (i.e., digit span, Stroop) (see Heath et al. 2016; Kaufman et al. 2012; Peltsch et al. 2014).

In terms of research predictions, hypoxic intervals were predicted to decrease SaO_2_ and ScO_2_ and increase ventilation, HR and CBF (i.e., MCAv). As such, it was hypothesized that an IH‐based increase in CBF would provide an immediate (T1) and delayed (T2) post‐protocol reduction in antisaccade RTs; that is, we predicted a post‐protocol EF benefit. Moreover, because an increase in CBF is thought to represent a primary moderator for an EF benefit, a secondary goal of this work was to identify whether hypoxic interval changes in CBF magnitude correlated with the magnitude of post‐protocol antisaccade RT benefits.

## 2 Methods

### 2.1 Participants

Twenty‐four healthy individuals (14 female and 10 male) ranging in age from 18 to 35 years (average = 22.7, SD = 4.1) from the University of Western Ontario community were recruited for this study. Sample size was determined a priori via G*Power based on Tari et al.'s (2020) protocol contrasting pre‐ and post‐hypercapnic (10‐min inhalation of 5% CO_2_) changes in antisaccade RTs (*d*_z_ = 0.61, power = 0.80, *α* = 0.05). Inclusion criteria required that participants had normal or corrected‐to‐normal vision; were not pregnant; had no history of smoking and/or cardiorespiratory, metabolic, musculoskeletal, neurological, or neuropsychiatric disorder; and were not taking medication impacting metabolic, cardiac, or hemodynamic responses to a hypoxic environment or to exercise. Moreover, because fitness influences cerebrovascular changes to environmental stressors, it was required that participants complete the Godin Leisure Time Exercise Questionnaire (GLTEQ) (Godin 2011). The average GLTEQ score was 60.2 (SD = 22.6; range = 26–120) and indicated that all participants were recreationally active. Participants were instructed to refrain from alcohol, caffeine, recreational drugs, and strenuous exercise 12 h prior to any study intervention and to get 8 h of sleep on the night prior to each study intervention. All participants reported adhering to these study requirements. Data collection took place between 9:30 am and 11:30 am with participants in a hydrated state (i.e., ~500 mL of water consumed 1‐h in advance of data collection).

Participants read a letter of information and provided informed written consent via a protocol approved by the Health Sciences Research Ethics Board, University of Western Ontario (#125185). This study conformed to the ethical standards of the most recent iteration of the Declaration of Helsinki with the exception that participants were not registered in a database.

### 2.2 Experimental Overview

**FIGURE 1:**
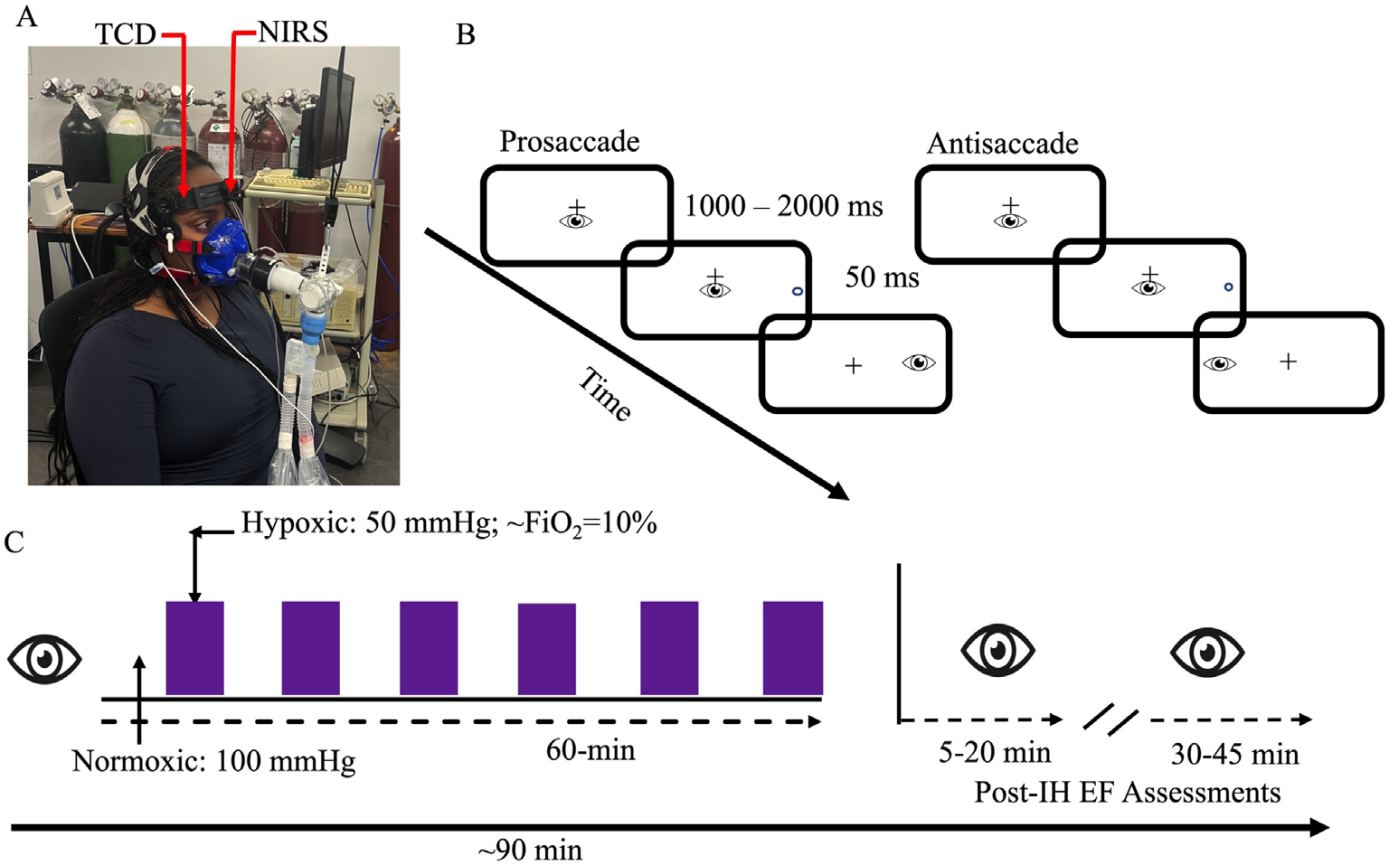
Panel A shows the experimental apparatus and placement for transcranial Doppler ultrasound and near‐infrared probes. Panel B shows the timeline of visual and motor events associated with pro‐ and antisaccade trials. Panel C provides a schematic of the timing of oculomotor assessments (i.e., eye icon) and the timing of the normoxic and hypoxic intervals within the intermittent hypoxia (IH) protocol. For all participants, the first interval in the IH protocol was normoxic and the last interval was hypoxic.

Participants completed IH (i.e., P_ET_O_2_ = 50 mmHg) and control (P_ET_O_2_ = 100 mmHg) protocols in a normobaric environment (251 m above sea level; ambient air temp 20°C–22°C) ordered via a Latin square and completed on different days separated by at least 48 h. For both protocols, participants sat for 20 min on a padded semi‐recumbent chair to account for the locomotor costs of arriving to the lab. Subsequently, participants completed a baseline EF assessment (T0) followed by the IH or control protocol. As shown in Figure 1, the IH protocol entailed 12 alternating 5‐min normoxic and hypoxic intervals (60‐min duration), whereas the control protocol entailed breathing normoxic air for 60 min. For the IH protocol, a normoxic interval was delivered first to record baseline physiological parameters (see details below). EF was again assessed immediately (T1) and 30 min (T2) following each protocol.

### 2.3 Apparatus and Procedures

For IH and control protocols, participants breathed through a face mask (7450 Series V2 Oro‐Nasal Reusable Face Mask; Hans Rudolph, Shawnee, KS, USA) connected in series to a turbine system (Universal Ventilation Meter bi‐directional; VacuMed; Ventura, CA, USA) that included a 3‐way T‐shaped valve and was used to measure respiratory flows (Figure 1). The second port on the 3‐way valve was open to room air, whereas the third port was connected to a sequential gas delivery circuit. The sequential gas delivery circuit was comprised of a non‐rebreathing valve, an expiratory gas reservoir, and an inspiratory gas reservoir. This setup permitted the investigator to manually switch participants between breathing gas mixtures with P_ET_O_2_ of 100 mmHg (i.e., normoxic) and 50 mmHg (i.e., hypoxic). Participants were kept at normocapnic levels (determined from the first normoxic interval) during control and IH protocols by administering a continuous flow of appropriate gas volume with composition that was controlled by a gas blender (GSM 3, CEW Inc., Ardmore, USA). In particular, the P_ET_CO_2_ measured during the first normoxic interval was maintained throughout the ensuing 5‐min intervals across IH and control protocols (i.e., normocapnic) and thus served to provide an isocapnic protocol. Moreover, by using the sequential gas delivery circuit to control participants' alveolar ventilation and PaCO_2_, our protocol did not require CO_2_ supplementation because the flow of fresh air to the participant was manipulated by altering the volume of rebreathed gas (i.e., lowering fresh gas inflow increases rebreathing per breath and increases P_ET_CO_2_; for review see, Fisher et al. 2016). Participants were instructed to breathe normally and freely in both protocols. Respired air was sampled continuously at the mouth via a sampling line (VacuMed, Ventura, CA, USA) and analyzed for fractional concentrations of O_2_ and CO_2_ (series 17500; VacuMed; Ventura, CA, USA). Flow and fractional concentration data were recorded at 100 Hz via a 16‐bit analog‐to‐digital converter. Custom software (LabView, National Instruments; Austin, TX, USA) (Guluzade et al. 2022) aligned gas concentrations and integrated flow signals and executed a peak‐detection algorithm to determine breath‐by‐breath pressures of end‐tidal CO_2_ (P_ET_CO_2_) and O_2_ (P_ET_O_2_), tidal volume, breathing frequency (B*f*), and minute ventilation (V̇_E_). HR and SaO_2_ were continuously measured with a pulse oximeter attached to the participant's earlobe (Model 7500, Nonin Medical Inc.; Plymouth, MN, USA). Prior to data collection, all systems were calibrated using room air and a precision‐mixed cylinder of known concentrations, and turbine volume was calibrated with a 3‐L syringe. For both testing sessions, we did not inform participants whether they were completing an IH or control protocol (i.e., single blind paradigm) and within the IH protocol participants could not see the experimenter manipulate the three‐way valve circuit that alternated between normoxic and hypoxic gas mixtures.

### 2.4 Cortical Hemodynamics

TCD (Neurovision TOC2M; Multigon Industries, Elmsford, CA, USA) and NIRS (Oxiplex TS, model 92505: ISS, Champaign, IL, USA) probes measured MCAv and ScO_2_, respectively. TCD (100 Hz) and NIRS (50 Hz) probes were placed on the right anterior temporal window and left frons, respectively, and were secured via an adjustable headband (Figure 1). The TCD probe was coated in an aqueous ultrasound gel (Aquasonic Clear, Parker Laboratories Inc., Fairfield, NJ, USA).

### 2.5 Oculomotor Executive Function

Participants sat on an adjustable chair in front of a table on which an LCD monitor (60 Hz, 8‐ms response rate, 1280 × 960 pixels; Dell 3007WFP, Round Rock, TX, USA) was located 550 mm from the table's front edge. For this assessment, participants placed their head in a head‐chin rest and the gaze location of their left eye was tracked via a video‐based eye tracking system (EyeLink 1000 Plus; SR Research, Ottawa, ON, Canada) sampling at 1000 Hz. Prior to data collection, a nine‐point calibration and validation were completed (i.e., < 1° of error). Experimental events were controlled via MATLAB (R2018a; The MathWorks, Natick, MA, USA) and the Psychophysics Toolbox extensions (v. 3.0) (Brainard 1997; Kleiner et al. 2007) including the EyeLink Toolbox (Cornelissen et al. 2002). The lights in the experimental suite provided 16 cd/m^2^ of ambient luminance.

Visual stimuli were presented on a black screen (0.1 cd/m^2^) and included a white midline‐located fixation cross (1°: 50 cd/m^2^) presented at participants' eye level and white targets (i.e., open circle; 2.5° in diameter: 127 cd/m^2^) located 13° (i.e., proximal target) and 16° (i.e., distal target) to the left and right of fixation and in the same horizontal plane. Fixation onset signaled participants to direct their gaze to its location. Once a stable gaze was achieved (i.e., ±1.5° for 450 ms), a uniformly distributed randomized foreperiod (1000–2000 ms) (via MATLAB “Rand” function) was introduced after which one of the targets appeared for 50 ms and cued participants to saccade to the veridical (i.e., prosaccade) or mirror‐symmetrical (i.e., antisaccade) target location “as quickly and accurately as possible”. The fixation cross remained visible for the duration of a trial (i.e., overlap paradigm). Pro‐ and antisaccades were completed in separate and randomly ordered blocks involving 20 pseudo‐randomly presented trials to each target location (i.e., left and right visual field) and eccentricity (i.e., proximal and distal). Oculomotor assessments were completed prior to IH and control protocols (T0) and ~5‐ (T1) and 30‐min (T2) following the cessation of each protocol. Each oculomotor assessment required approximately 15 min to complete. Notably, antisaccades provide “excellent” internal consistency and reliability at baseline and retest (Weiss and Luciana 2022) and exhibit “high test–retest” reliability over multiple weeks of repeated testing (Klein and Berg 2001; Weiler et al. 2015). Hence, the task was unlikely to have been influenced by practice‐related performance benefits over the course of the oculomotor assessments used here. Moreover, by contrasting pro‐ and antisaccades, our study provides a paradigm to determine whether a protocol‐induced change in oculomotor performance reflects a general change to information processing (i.e., a pro‐ and antisaccade benefit) or a change specific to high‐level EF (i.e., a selective antisaccade benefit).

### 2.6 Data Reduction, Dependent Variables and Statistical Analyses

For respiratory, cardiovascular, TCD, and NIRS measures, data points ±3 standard deviations from a participant‐specific mean were removed (Lamarra et al. 1987), and for the former two measures, data corrupted by signal aliasing and/or signal loss (i.e., a sudden head shift) were omitted (see Terslev et al. 2017). Respiratory data were linearly interpolated on a second‐by‐second basis, time‐aligned to the onset of a protocol interval and/or session, and averaged into 5 s bins (Keir et al. 2015).

For the oculomotor task, gaze position data were filtered via a dual‐pass Butterworth filter with a low‐pass cutoff frequency of 15 Hz, and instantaneous velocities were computed using a five‐point central finite difference algorithm. Acceleration data were similarly obtained from the velocity. Saccade onset was marked when velocity and acceleration exceeded 30°/s and 8000°/s^2^, respectively, and was confirmed interactively via trial‐by‐trial inspection. Saccade offset was marked when saccade velocity was below 30°/s for 40 ms. Trials involving signal loss (e.g., eye blink) were excluded, as were trials with an amplitude < 2° (Weiler and Heath 2014) and/or a RT less than 50 ms or ±3 standard deviations from a participant‐specific mean (Wenban‐Smith and Findlay 1991). Less than 6% of trials were removed for any participant.

Dependent variables for physiological measures included HR, SaO_2_, ScO_2_, P_ET_O_2_, P_ET_CO_2_, V̇_E_, breathing frequency (B*f*), and peak systolic MCAv, with each metric averaged over 5 min (i.e., the time associated with each interval). Mean arterial pressure (MAP) was measured at a discrete point during each interval, and this and mean MCAv were used to compute a cerebrovascular conductance index (CVCi = MCAv/MAP). Oculomotor dependent variables included RT (i.e., time from response cueing to saccade onset), saccade duration (i.e., time from saccade onset to offset), saccade gain variability (i.e., within‐participant standard deviation of saccade amplitude/veridical target location), and directional errors (i.e., a prosaccade instead of an instructed antisaccade or vice versa).

Each dependent variable met the assumption of normality (Q‐Q plots and Shapiro–Wilk test) and homogeneity (Levene's test) and were evaluated for violations of sphericity (Mauchly's *p*s < 0.05)—and where appropriate—Huynh‐Feldt corrected degrees of freedom are reported to one decimal place. For respiratory, cardiovascular, and cortical hemodynamic data, separate ANOVA models were used for control and IH protocols. For the control protocol, data were examined via one‐way (i.e., interval: 1, 2, …, 11, 12) repeated measures ANOVAs, whereas IH protocol data were examined via 2 (state: normoxic, hypoxic) by 6 (interval: 1, 2, … 5, 6) fully repeated measures ANOVAs. The different ANOVA models were necessary given that the control condition did not include an interval‐based change in the composition of the inhaled gas mixture (i.e., normoxic vs. hypoxic). For the oculomotor variables, data were examined via 2 (protocol: normoxic, hypoxic) by 3 (time: T0, T1, T2) by 2 (task: pro‐, antisaccade) fully repeated measures ANOVAs. For all ANOVAs, significance was determined at *α* ≤ 0.05, and significant main effects and interactions were decomposed via simple effects and/or power polynomials (i.e., trend analysis) (Pedhazur 1977). For control protocol respiratory, cardiovascular and cortical hemodynamic variables, one‐way Bayesian ANOVAs (BF_01_) were used to test the null hypothesis. For the analyses of oculomotor RT data, Bayesian paired‐samples t‐tests (Cauchy distribution = 0.707) evaluating the alternative hypothesis (i.e., BF_10_) were used to complement frequentist statistics. Jeffreys' (1961) nomenclature of “anecdotal” (i.e., 1 to < 3), “moderate” (i.e., 3 to < 10), “strong” (i.e., 10 to < 100) and “very strong” (i.e., > 100) was used to contextualize Bayes factor robustness.

## 3 Results

**FIGURE 2:**
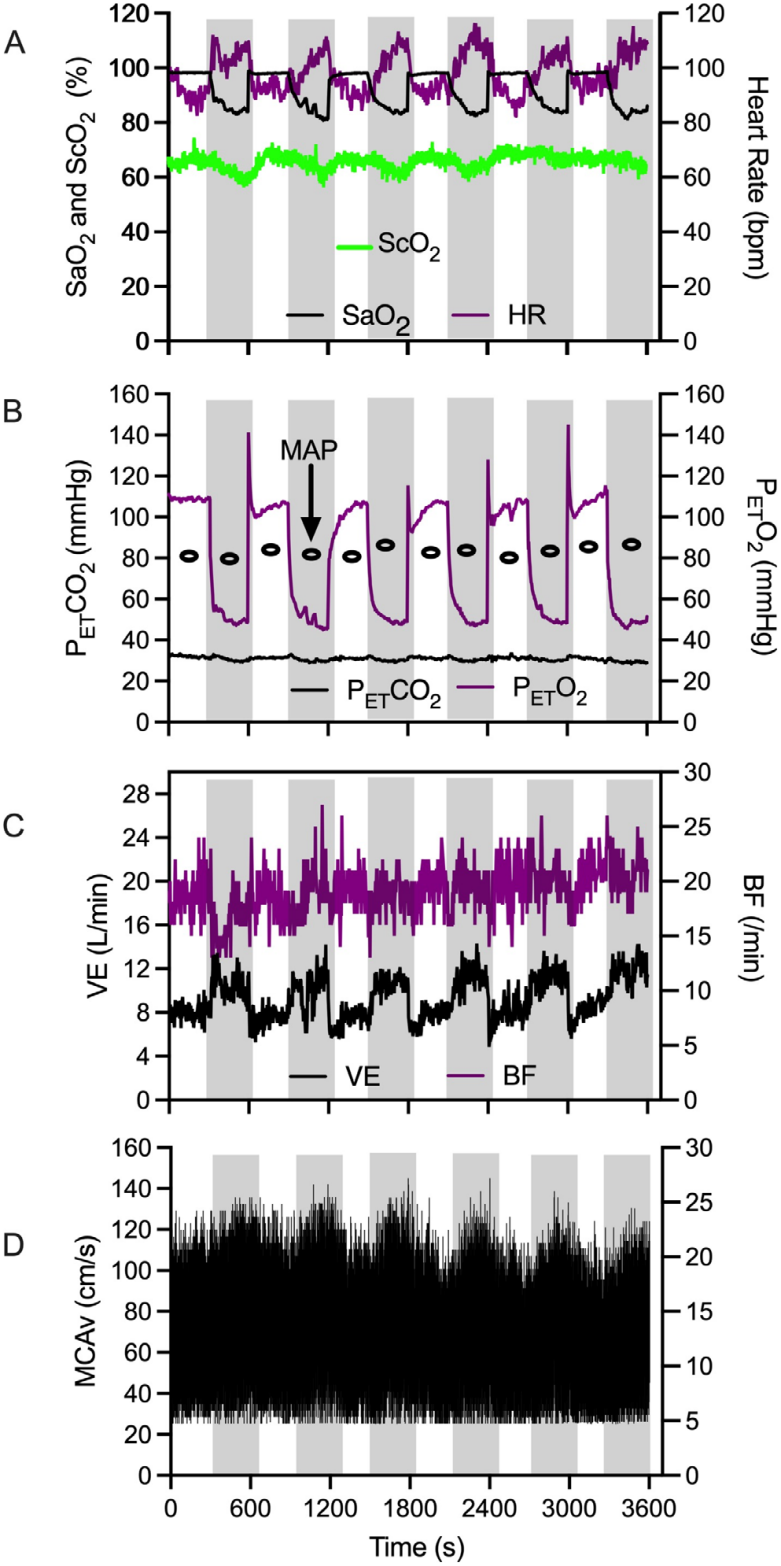
Data for an exemplar participant showing continuous: (1) Panel A: Arterial O_2_ saturation (SaO_2_), cerebral tissue oxygenation (ScO_2_) and heart rate (HR), (2) Panel B: End‐tidal CO_2_ (P_ET_CO_2_) and end‐tidal O_2_ (P_ET_O_2_), (3) Panel C: Minute ventilation (V̇_E_) and breathing frequency (B*f*), (4) Panel D: Middle cerebral artery velocity (MCAv), as a function of successive normoxic and hypoxic intervals. For this figure, hypoxic intervals are denoted via light gray shading. Panel B provides mean arterial pressure (MAP) at discrete timepoints in normoxic and hypoxic intervals.

Figure 2 presents respiratory, cardiovascular, and cortical hemodynamic data for an exemplar participant and demonstrates a decrease in SaO_2_, ScO_2_, P_ET_O_2_, and B*f* and an increase in HR, V̇_E_, and MCAv during hypoxic intervals. The exemplar participant did not demonstrate a change in P_ET_CO_2_ or MAP across normoxic and hypoxic intervals. In terms of quantitative analyses, below, we outline respiratory, cardiovascular, and cortical hemodynamic data separately for the control and IH conditions.

### 3.1 Respiratory and Cardiovascular Variables

#### 3.1.1 Control Condition

**FIGURE 3:**
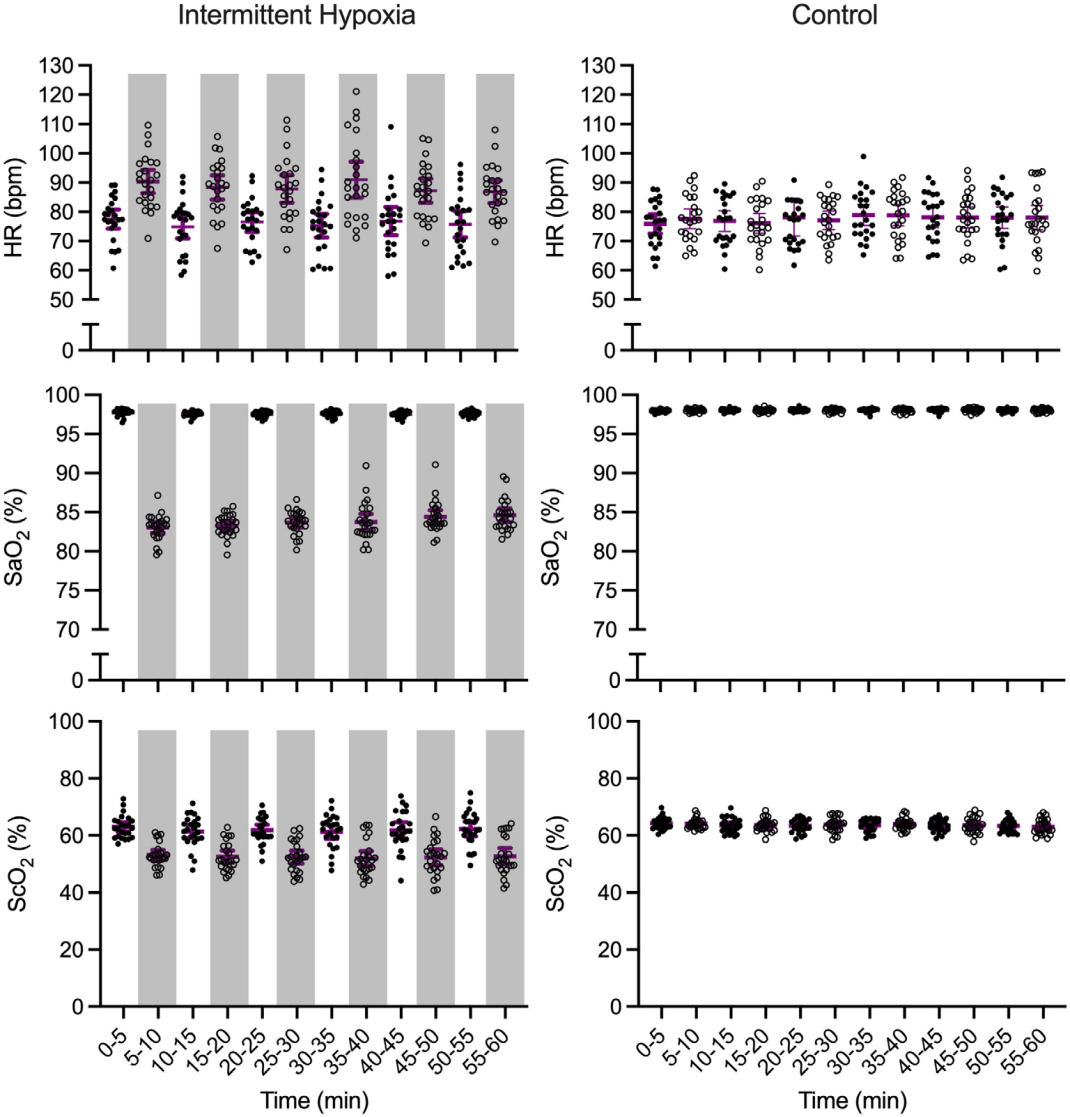
Participant‐specific and mean (purple bar) heart rate (HR), arterial O_2_ saturation (SaO_2_) and cerebral tissue oxygenation (ScO_2_) for intermittent hypoxia and control protocols across successive intervals. For the intermittent hypoxia protocol, hypoxic intervals are denoted via light gray shading. Error bars represent 95% between‐participant confidence intervals.

**FIGURE 4:**
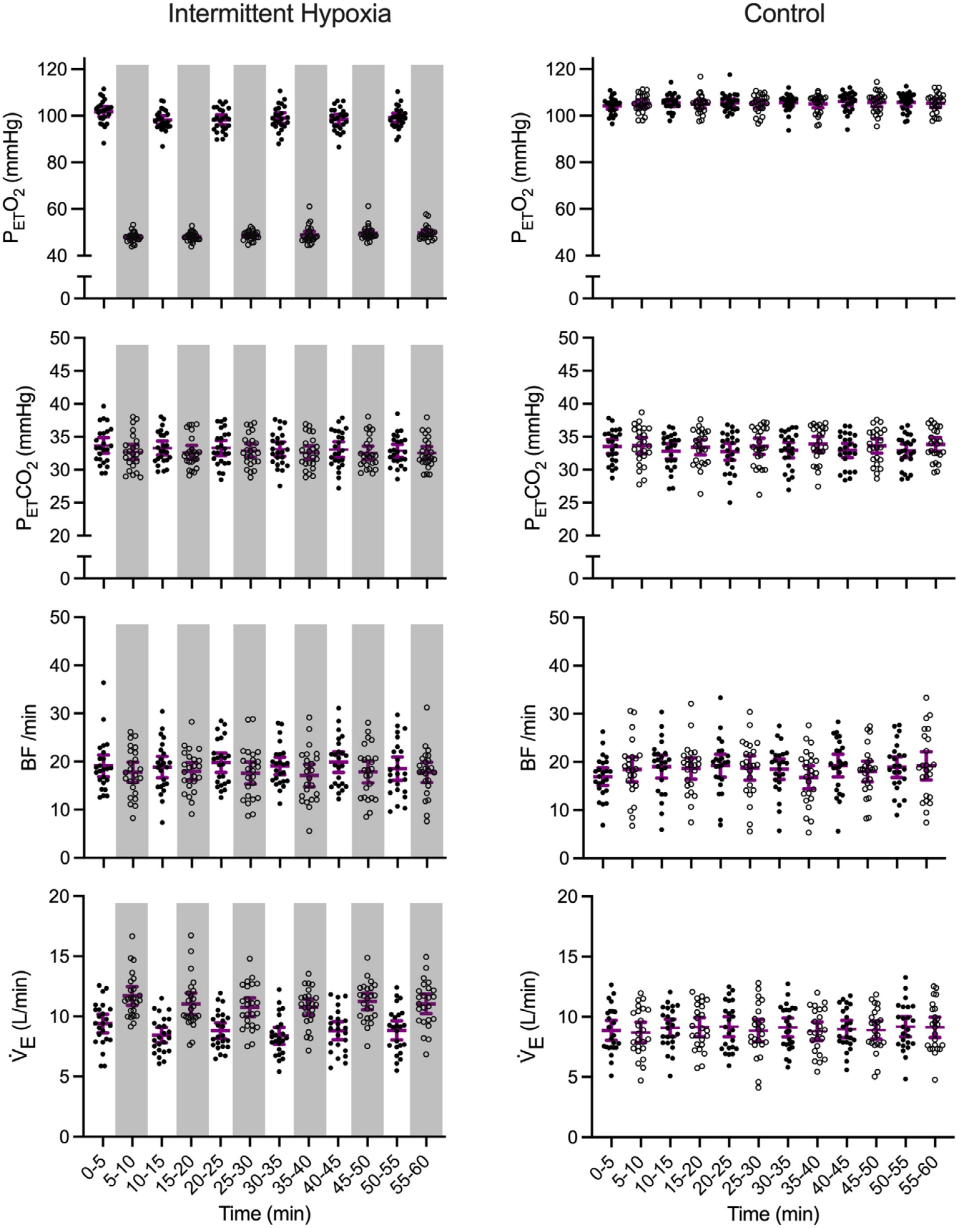
Participant‐specific and mean (purple bar) end‐tidal CO_2_ (P_ET_CO_2_), end‐tidal O_2_ (P_ET_O_2_), minute ventilation (V̇_E_) and breathing frequency (B*f*) for intermittent hypoxia and control protocols across successive intervals. For the intermittent hypoxia protocol, hypoxic intervals are denoted via light gray shading. Error bars represent 95% between‐participant confidence intervals.

The ANOVA summary statistics for these variables are provided in Table 1. Figures 3 and 4 demonstrate that results did not produce main effects of interval (*p*s > 0.47), and for all variables one‐way Bayesian ANOVAs indicated moderate to strong evidence favoring the null hypothesis (i.e., all BF_01_ > 5.53–68.67).

**TABLE 1:** Control protocol respiratory, cardiovascular and cortical hemodynamic data ANOVA summary statistics. Hunyh‐Feldt corrected degrees of freedom are reported to one decimal place.

Variable	df	*F*	*p*	$\eta_{p}^{2}$
SaO_2_: *Interval*	5.4, 124.2	0.81	=0.629	0.03
ScO2: *Interval*	8.6, 198.8	1.25	=0.264	0.05
P_ET_O_2_: *Interval*	4.9, 112.8	0.89	=0.484	0.03
P_ET_CO_2_: *Interval*	5.1, 118.3	1.03	=0.234	0.04
V̇_E_: *Interval*	6.8, 156.8	0.99	=0.433	0.04
B*f*: *Interval*	8.7, 202.2	1.79	=0.073	0.07
HR: *Interval*	3.0, 66.5	1.29	=0.284	0.05
MAP: *Interval*	8.5, 196.0	1.80	=0.074	0.07
MCAv: *Interval*	3.8, 89.1	0.86	=0.488	0.03
CVCi: *Interval*	5.5, 127.9	1.86	=0.097	0.07

Abbreviations: B*f*, breathing frequency; CVCi, cerebrovascular conductance index; HR, heart rate; MAP, mean arterial pressure; MCAv, middle cerebral artery velocity; P_ET_O_2_ and P_ET_CO_2_, end‐tidal O_2_ and CO_2_, respectively; SaO_2_, arterial saturation; ScO_2_, cerebral tissue saturation; V̇_E_, minute ventilation.

#### 3.1.2 Intermittent Hypoxia (IH) Condition

P_ET_O_2_, P_ET_CO_2_, SaO_2_, V̇_E_, B*f*, and HR elicited main effects of state (*p*s < 0.02) (see Table 2 for ANOVA summary statistics). SaO_2_ (Figure 3), P_ET_O_2_, P_ET_CO_2_, and B*f* (Figure 4) values were decreased during hypoxic compared to normoxic intervals. For P_ET_O_2_, SaO_2_, and B*f*, the average decrease from normoxic to hypoxic states was 50 mmHg (SD = 5), 14% (SD = 1), and 1.5 (breath/min: SD = 4), respectively, whereas the P_ET_CO_2_ decrease was 0.5 mmHg (SD = 1). In turn, HR (Figure 3) and V̇_E_ (Figure 4) values were greater during hypoxic than normoxic intervals. As well, P_ET_O_2_ and SaO_2_ elicited main effects of interval (*p*s < 0.03) and interval by state interactions (*p*s < 0.01) (see Table 2). Paired‐samples *t*‐tests for both variables showed that values were decreased for hypoxic than normoxic states at each successive interval (*p*s < 0.001) and thus indicated the magnitude of the between‐state difference varied as a function of interval. Accordingly, we computed participant‐specific P_ET_O_2_ and SaO_2_ difference scores (normoxic minus hypoxic) separately for each interval and evaluated via power‐polynomials. Results showed that difference scores decreased linearly as a function of increasing interval (only linear effects significant: *F*s(1, 23) = 8.90 and 47.80 for P_ET_O_2_ and SaO_2_, respectively, *p*s < 0.001),^1^ such that P_ET_O_2_ and SaO_2_ decreased by 4 mmHg (SD = 2) and 2% (SD = 1), respectively, from the first to last interval.

**TABLE 2:** Intermittent hypoxia protocol respiratory, cardiovascular and cortical hemodynamic data ANOVA summary statistics. Where appropriate, Hunyh‐Feldt corrected degrees of freedom are reported to one decimal place.

Variable	df	*F*	*p*	$\eta_{p}^{2}$
SaO_2_				
Interval	2.8, 66.2	3.29	0.027	0.12
State	1, 23	2694.08	< 0.001	0.99
Time by state	2.8, 65.8	3.68	0.018	0.14
ScO_2_				
Interval	3.1, 71.3	1.34	0.249	0.05
State	1, 23	153.24	< 0.001	0.87
Time by state	5, 115	0.32	0.90	0.01
P_ET_O_2_				
Interval	4.0, 93.1	3.48	0.010	0.13
State	1, 23	2754.41	< 0.001	0.99
Time by state	3.7, 86.2	5.188	< 0.001	0.18
P_ET_CO_2_				
Interval	4.1, 95.5	1.65	0.166	0.06
State	1, 23	7.24	0.013	0.24
Time by state	5, 115	1.70	0.136	0.06
V̇_E_				
Interval	3.2, 73.7	1.38	0.234	0.05
State	1, 23	102.80	< 0.001	0.82
Time by state	5, 115	2.85	0.061	0.09
B*f*				
Interval	4.5, 105.1	0.54	0.729	0.02
State	1, 23	7.54	0.012	0.24
Time by state	5, 115	0.55	0.716	0.02
HR				
Interval	3.1, 73.0	2.62	0.054	0.10
State	1, 23	256.25	< 0.001	0.92
Time by state	2.4, 56.7	2.19	0.110	0.08
MAP				
Interval	3.8, 88.0	2.10	0.072	0.08
State	1, 23	0.06	0.804	< 0.01
Time by state	5, 115	1.37	0.246	0.05
MCAv				
Interval	3.5, 81.6	6.20	< 0.001	0.21
State	1, 23	16.60	< 0.001	0.42
Time by state	3.7, 87.2	3.01	0.024	0.11
CVCi				
Interval	5, 115	8.56	< 0.001	0.27
State	1, 23	12.22	0.002	0.38
Time by state	5, 115	2.34	0.046	0.09

Abbreviations: B*f*, breathing frequency; CVCi, cerebrovascular conductance index; HR, heart rate; MAP, mean arterial pressure; MCAv, middle cerebral artery velocity; P_ET_O_2_ and P_ET_CO_2_, end‐tidal O_2_ and CO_2_, respectively; SaO_2_, arterial saturation; ScO_2_, cerebral tissue saturation; V̇_E_, minute ventilation.

The average MAP for normoxic and hypoxic states was 86 mmHg (SD = 7) and 86 mmHg (SD = 7), respectively, and this variable did not produce main effects or interactions (*p*s > 0.80).

### 3.2 Cortical Hemodynamic Variables

#### 3.2.1 Control Condition

**FIGURE 5:**
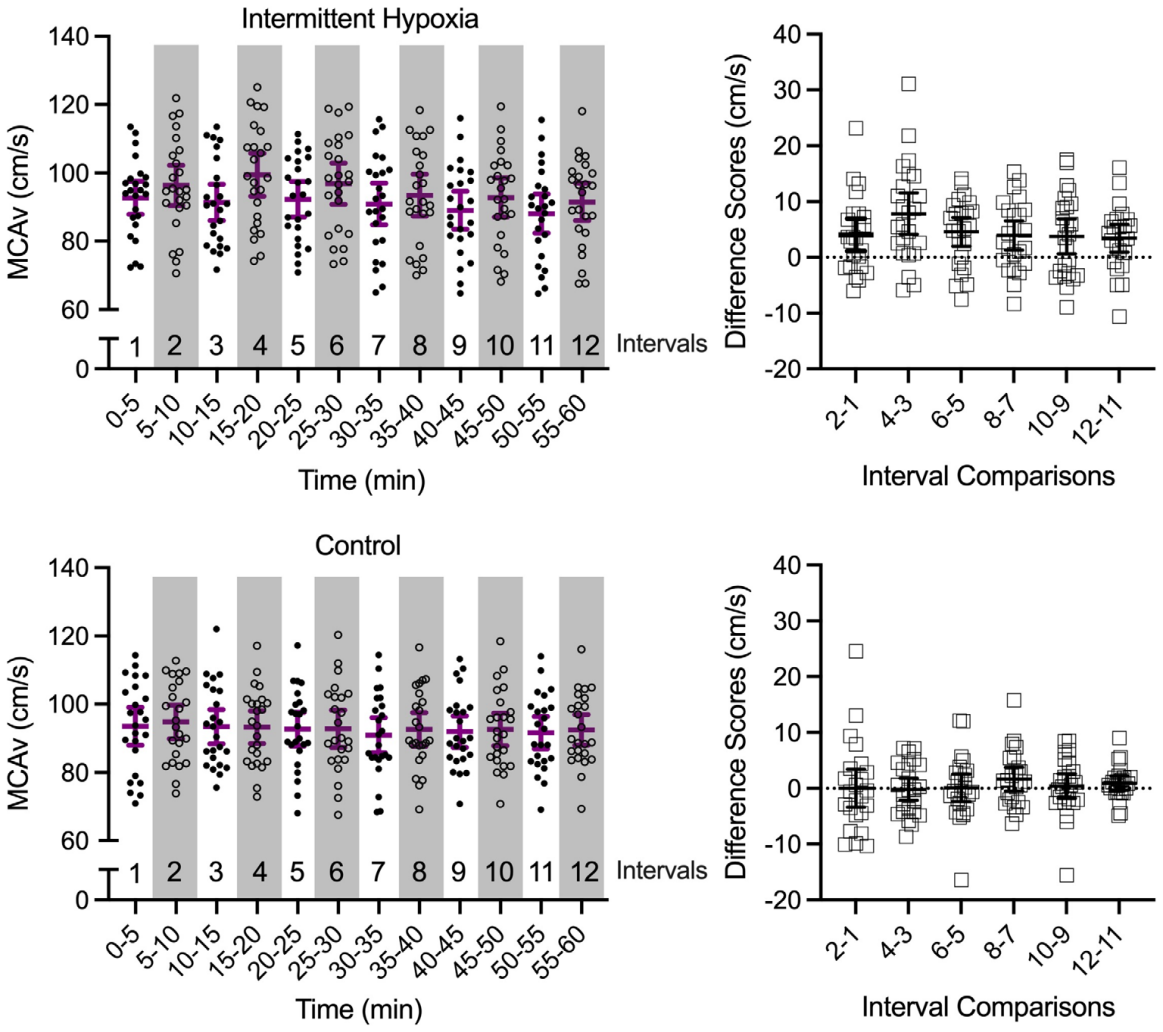
The large main panels show participant‐specific and mean (purple bar) middle cerebral artery velocity (MCAv) for intermittent hypoxia and control protocols across successive intervals. For the intermittent hypoxia protocol, hypoxic intervals are denoted via light gray shading. Within the figure the numerical values above the x‐axis indicate successive 5‐min interval numbers. The smaller offset panels show MCAv difference scores (hypoxic minus normoxic intervals) across successive intervals (i.e., interval 2 vs. interval 1, …, interval 12 minus interval 11) for the intermittent hypoxia protocol, and between successive normoxic intervals for the control protocol. Error bars represent 95% between‐participant confidence intervals and for the smaller offset panels, the absence of overlap between an error bar and zero (horizontal dotted line) represents a reliable difference inclusive to a test of the null hypothesis.

Figures 3 and 5 demonstrate that ScO_2_, MCAv, and CVCi did not produce significant main effects of interval (*p*s > 0.25) (Table 1) with Bayesian ANOVAs indicating strong support for the null hypothesis (BF_01_ > 16.10–58.84).

#### 3.2.2 Intermittent Hypoxia (IH) Condition

SaCO_2_, MCAv, and CVCi produced main effects of state (*p*s < 0.011) (Table 2). SaCO_2_ decreased on average by 14% (SD = 1) from normoxic to hypoxic intervals, whereas MCAv and CVCi increased by 7 cm/s (SD = 7) and 0.05 cm/s/mmHg (SD = 0.11), respectively, from control to hypoxic intervals. Last, MCAv (Figure 5) and CVCi produced main effects of interval (*p*s < 0.001) and state‐by‐interval interactions (*p*s < 0.05) indicating that values were larger for hypoxic than normoxic states at each interval (*p*s < 0.001). As described above (see P_ET_O_2_ and SaO_2_), to further explore these interactions we computed MCAv and CVCi difference scores (normoxic minus hypoxic) and observed that both variables decreased linearly from the first to last interval (only linear effects significant: *F*(1, 23) = 7.85 and 9.35 for MCAv and CVCi, respectively, *p*s < 0.001).

### 3.3 Oculomotor Variables

#### 3.3.1 Reaction Time

**FIGURE 6:**
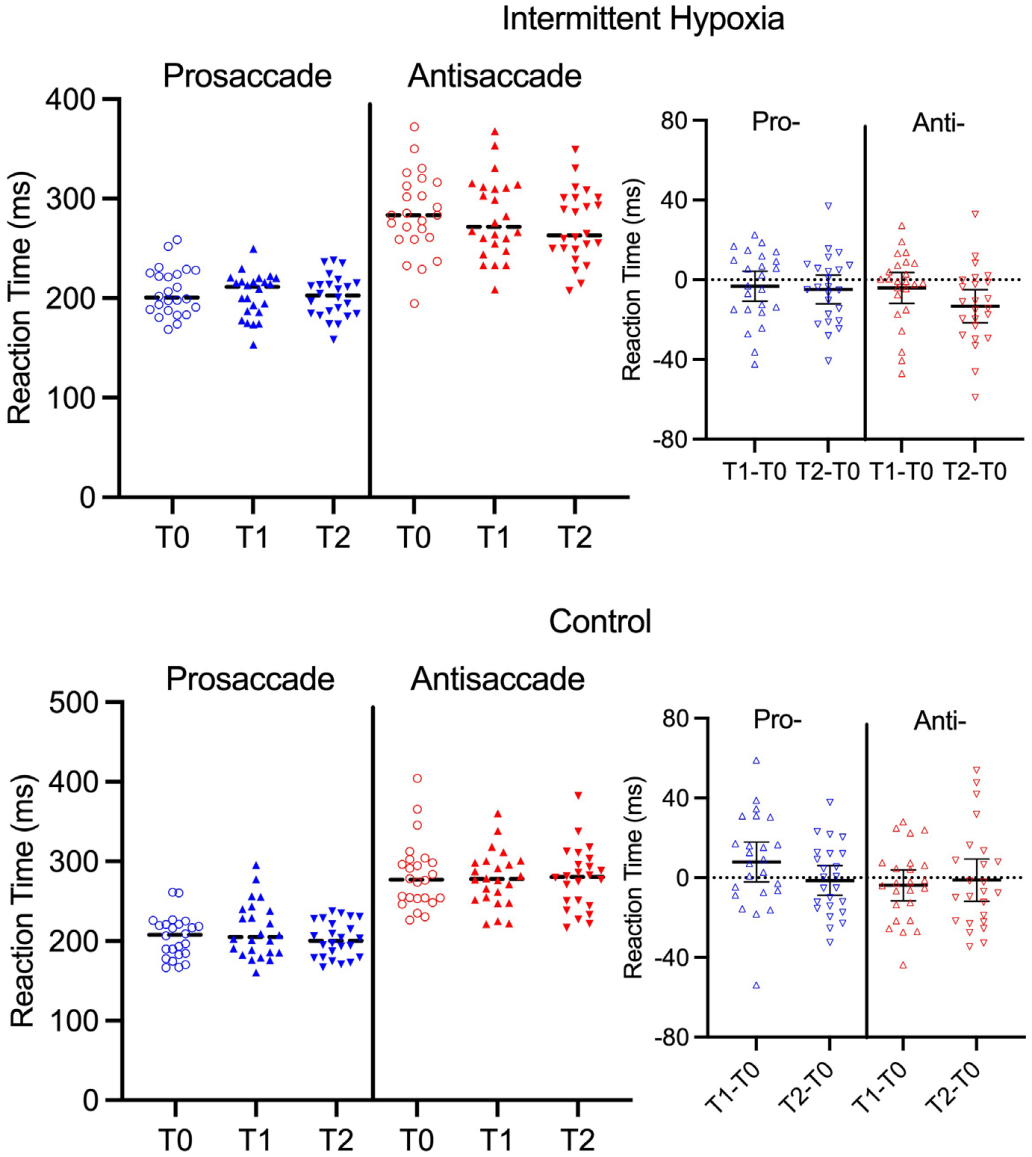
The large main panels show participant‐specific and mean (see black bar) pro‐ (blue symbols) and antisaccade (red symbols) reaction time for intermittent hypoxia and control protocols at pre‐ (T0) and immediate (T1) and 30‐min (T2) post‐protocol assessments. The smaller offset panels represent participant‐specific and mean pro‐ and antisaccade reaction time difference scores (i.e., T1 minus T0; T2 minus T0). Error bars represent 95% between‐participant confidence intervals and for the smaller offset panels, the absence of overlap between an error bar and zero (horizontal dotted line) represents a reliable difference inclusive to a test of the null hypothesis.

Results yielded main effects for time, *F*(2, 46) = 3.67, *p* = 0.033, $\eta_{p}^{2}$ = 0.14, task, *F*(1, 23) = 135.53, *p* < 0.001, $\eta_{p}^{2}$ = 0.85, and a protocol by time by task interaction, *F*(2, 46) = 3.64, *p* = 0.034, $\eta_{p}^{2}$ = 0.14. Prosaccade RTs (205 ms, SD = 26) were shorter than antisaccades (276 ms, SD = 40) – a finding independent of protocol and time of assessment. To decompose the interaction, we computed participant‐specific pro‐ and antisaccade RT difference scores (T1 minus T0; T2 minus T0) separately for control and IH protocols and examined via single‐sample t‐statistics. The offset panels of Figure 6 show that prosaccade difference scores for control (*t*s(23) = 1.33 and −0.37, for T1 and T2 respectively, *p*s > 0.19, all *d*_z_ < |0.27|) and IH (*t*s(23) = −0.90 and −1.38, *p*s > 0.18, all *d*_z_ < |0.28|) protocols did not reliably differ from zero. In turn, antisaccade difference scores in the control protocol did not differ from zero at T1 or T2 (*t*s(23) = −1.01 and −0.22, respectively, *p*s > 0.32, all *d*_z_ < |0.28|), and for the IH protocol values did not differ from zero at T1 (*t*(23) = −1.08, *p* = 0.29, *d*_z_ = −0.22). In contrast, antisaccade difference scores for the IH protocol at T2 were less than zero (*t*(23) = −3.24, *p* = 0.004, *d*_z_ = −0.62). As well, Bayesian single‐sample t‐statistics for IH condition antisaccade RT difference scores at T2 (BF_10_ = 11.05) showed “strong” support for the alternate hypothesis, whereas all other difference score BF_10_ values were less than 0.65 and thus provide no evidence supporting the alternate hypothesis. Thus, frequentist and Bayesian statistics showed a selective reduction in IH protocol antisaccade RTs at T2 and this difference was characterized by a “moderate” effect size benchmark.

#### 3.3.2 Directional Errors

Results revealed a main effect for task, *F*(1, 23) = 21.13, *p* < 0.001, $\eta_{p}^{2}$ = 0.47: directional errors were less for pro‐ (3%, SD = 4) than for antisaccades (10%, SD = 8). We did not observe a main effect of protocol nor higher‐order interactions involving protocol, *F*s < 1, *p*s > 0.84, all $\eta_{p}^{2}$ < 0.01.

#### 3.3.3 Saccade Duration and Gain Variability

Results yielded main effects of task, *F*s(1, 23) = 5.57 and 133.86 for saccade duration and gain variability, respectively, *p*s = 0.027 and < 0.001, $\eta_{p}^{2}$ = 0.20 and 0.85. Prosaccades had shorter durations (53 ms, SD = 7) and less variable saccade gains (0.12, SD = 0.03) than antisaccades (saccade duration: 59 ms, SD = 13; gain variability: 0.22, SD = 0.05).

### 3.4 Correlation Between Respiratory, Cardiovascular and Cortical Hemodynamic Variables With Antisaccade RT Difference Scores

We correlated antisaccade RT difference scores at T2 (i.e., T2 minus T0) with selected respiratory (i.e., P_ET_O_2_, B*f*), cardiovascular (i.e., SaO_2_), and cortical hemodynamic (ScO_2_, MCAv, CVCi) measures. Results did not produce any significant correlation (*p*s > 0.17) and demonstrates that a unitary physiological measure did not predict the T2 improvement in antisaccade RTs. As well, and given the primary objective of this work, Bayesian correlations involving ScO_2_ and MCAv with antisaccade RT difference scores produced BF_01_ values of 2.72 and 2.20, respectively, and thus demonstrate anecdotal support for null associations.

## 4 Discussion

We examined whether a single bout of IH provides a post‐intervention “boost” to EF and whether a putative benefit relates to an increase in CBF. In outlining our results, we discuss the respiratory, cardiovascular, and cortical hemodynamic responses to IH before discussing the impact on EF.

### 4.1 Respiratory, Cardiovascular and Cortical Hemodynamic Responses to Intermittent Hypoxia

Hypoxic intervals decreased SaO_2_, ScO_2_, and P_ET_O_2_ by 14%, 10%, and 50 mmHg, respectively, with a rapid rebound to baseline for all measures when participants transitioned to a normoxic interval. These reductions demonstrate the consequence of a P_ET_O_2_ of 50 mmHg (for review see Powell and Garcia 2000). As well, hypoxic intervals increased HR and V̇_E_ and represent chemoreceptive‐induced adjustments supporting homeostatic O_2_ delivery to the brain and other primary regulatory organs (Buchheit et al. 2004). In turn, the magnitude of the SaO_2_, ScO_2_, and P_ET_O_2_ differences between hypoxic and normoxic intervals decreased across successive intervals and is a result linked to an adaptive shift in the hemoglobin‐O_2_ dissociation curve to potentiate O_2_ availability (Peltonen et al. 2007; Zhang et al. 2010).

The reduction in ScO_2_ during hypoxic intervals was accompanied by an increase in MCAv and CVCi without a change in MAP. The hypoxic‐induced increase in MCAv observed here (7 cm/s, SD = 7) is consistent with previous work involving a single bout isocapnic protocol (6 cm/s) (AlSalahi et al. 2021) and a chronic poikilocapnic IH protocol (~9 cm/s) (Wang et al. 2020). These findings demonstrate that cerebrovascular dilation, and not a change in perfusion pressure, maintained O_2_ delivery to the brain during hypoxic intervals (Liu et al. 2017; but see Steinback and Poulin 2008). As well, MCAv and CVCi showed a decrease in the magnitude of the difference between hypoxic and normoxic intervals as a function of successive intervals and is a result human (for review see, Manukhina et al. 2016) and animal (Guan et al. 2023; Mashina et al. 2006) research has attributed to enhanced cortical O_2_ extraction following repeated exposure to brief hypoxic intervals. Thus, our findings demonstrate a protocol to examine whether a single bout of IH elicits a transient impact on EF.

We sought to maintain CO_2_ tension at a baseline level (i.e., normocapnic) and equated CO_2_ tension between hypoxic and normoxic intervals (i.e., isocapnic), whereas other work examining the impact of IH and EF did not (i.e., poikilocapnic) (Wang et al. 2020; Zhang et al. 2025). Notably, our hypoxic intervals produced an average 0.5 mmHg reduction in P_ET_CO_2_ compared to normoxic intervals. However, such a small reduction is within the normal breath‐to‐breath variability of P_ET_CO_2_ during spontaneous breathing and is unlikely to have lowered MCAv significantly given the slope of the MCAv—PCO_2_ relationship may be as low as 1% per mmHg of P_ET_CO_2_ at 12% FiO_2_ (Ogoh et al. 2014), and is likely a 6‐ to 8‐fold decrease in reduction compared to a poikilocapnic protocol (Zhang et al. 2014). Thus, the reported respiratory, cardiovascular and cortical hemodynamic data are in line with well‐documented physiological changes associated with an isocapnic IH protocol (for review see, Behrendt et al. 2022).

### 4.2 Antisaccade Metrics: Evidence for Top‐Down Executive Function

A general finding was that prosaccades produced shorter RTs and saccade durations, less variable gains, and decreased directional errors than antisaccades—a result consistent across IH and control protocols and for each oculomotor assessment (i.e., T0, T1, T2). The shorter prosaccade RTs and reduced directional errors reflect response mediation via direct retinotopic projections to the superior colliculus (Wurtz and Albano 1980) that operate largely independent of top‐down EF (Pierrot‐Deseilligny et al. 1995). In turn, the longer antisaccade RTs and increased directional errors indicate the time‐consuming—and sometimes error prone—EF demands of inhibiting a pre‐potent response (i.e., prosaccade) and decoupling the normally direct spatial relations between stimulus and response (Munoz and Everling 2004). Further, that antisaccades produced longer saccade durations and more variable gains reflects response mediation via visual information (i.e., relative) functionally distinct from the absolute visual information mediating prosaccades (Gillen and Heath 2014). Thus, the distinct metrics characterizing pro‐ and antisaccades provide a protocol for evaluating whether a single bout of IH impacts information processing (i.e., improvement in pro‐ and antisaccade metrics) or provides a selective benefit (or detriment) to EF (i.e., improvement in antisaccade—but not prosaccade—metrics).

### 4.3 Intermittent Hypoxia Produces a Selective Executive Function Benefit

Prosaccade metrics were not influenced across IH and control protocols and is a finding in line with work showing that exercise and other environmental stressors do not influence the planning of a pre‐potent oculomotor response (for review see, Zou et al. 2023). For antisaccades, IH protocol RTs did not differ between baseline (T0) and the immediate post‐protocol assessment (T1); however, at the 30‐min assessment (T2) RTs were reliably shorter than at T0 (i.e., 6% reduction). In contextualizing this finding, the T2 improvement could not be attributed to a practice‐related performance benefit given that the control protocol did not produce a similar change. Moreover, the RT benefit could not be attributed to an implicit—or explicit—speed‐accuracy trade‐off (Fitts 1954) because saccade duration, gain variability, and directional errors did not differ between timepoints (i.e., T0, T1, T2). Thus, a selective EF benefit was observed for a period beginning at 30‐min and extending up to 45‐min (i.e., the time from the start to end of T2) following the IH protocol.

At least two important issues require addressing. The first relates to the observation that the IH protocol did not produce an EF benefit at T1. This is salient because the exercise literature has shown that 11–20 min postexercise produces the largest and most reliable EF benefit (for meta‐analyses see, Chang et al. 2012) with more limited benefits observed up to 60 min (Hung et al. 2013; Joyce et al. 2009; Shukla and Heath 2022). The postexercise EF benefit is in part linked to an improvement in affect (e.g., enhanced mood) that facilitates attentional control in EF‐demanding tasks (Ayala and Heath 2021; Crush et al. 2018). In contrast, when transitioning from the partially reclined chair used in the IH protocol to the upright chair used for the oculomotor assessment, some participants reported the onset of somatic symptoms (i.e., headache, dizziness) that persisted for 5–10 min (for extensive review see, Burtscher et al. 2022).^2^ Thus, because the present investigation required participants to stand and transfer between the chairs used for the IH protocol and the oculomotor assessment (i.e., ~1.5 m away and requiring ~ two steps), the onset of symptomology (i.e., a “brain fog”) may have precluded an optimal level of task‐based attentional focus supporting the expression of an EF benefit. Thus, future work should employ a symptom scale (Rupp et al. 2013) to understand the potential linkage between post‐IH symptom burden and the manifestation of an EF benefit. The second issue relates to the mechanism(s) by which the IH protocol provided a T2 EF benefit. The most parsimonious account is that an increase in CBF and/or improved O_2_ extraction provided transient (i.e., 30–45 min) thermal, mechanical and/or biomolecular changes supporting EF network efficiency. For example, the CBF response to IH increases shear‐related endothelial stress (Iwamoto et al. 2020) and is a response that stimulates vasoactive substances (e.g., nitric oxide) supporting synaptic activity (Katusic et al. 2023). Moreover, animal models have shown that multiple IH sessions improve CBF and enhance mitochondrial structure and function supporting cognitive and motor abilities (Geary 2021; Mankovska and Serebrovska 2015). That said, frequentist and Bayesian correlation analyses demonstrated that our cortical hemodynamic variables (e.g., ScO_2_ and MCAv) were not related to the magnitude of the post‐IH reduction in antisaccade RTs. Although these results counter our a priori hypothesis, it may be that an EF benefit is not limited to a unitary physiological change; rather, the benefit may reflect interdependent changes in CBF and pressor responses (Washio and Ogoh 2023), biomolecule availability (e.g., nitric oxide), and enhanced functional connectivity within EF networks (Schmitt et al. 2019). Regardless of the explanation, the present results add importantly to the literature insomuch as they provide a first demonstration that a single bout of IH supports a transient EF “boost”.

### 4.4 Study Limitations

We recognize the generalizability of our study is limited by several methodological traits. First, we investigated the role of a single bout of IH on EF in healthy young adults. It is therefore unclear whether healthy older adults and/or individuals with hypoperfusion would demonstrate a similar benefit. Second, because our EF assessments were completed at immediate and 30‐min post‐protocol timepoints, we are unable to assert for how long an EF benefit may persist. Third, although antisaccades provide a directed measure of inhibitory control, the task does not quantify additional EF components (i.e., working memory and cognitive flexibility), and it is unclear whether a single bout of IH provides an encompassing EF benefit. Fourth, TCD does not quantify variations in vessel diameter when investigating changes in MCAv. This could be a potential limitation because the MCA may dilate or constrict in response to alterations in physiological conditions (Coverdale et al. 2015). Nonetheless, to the best of our knowledge, these changes do not compromise the validity of TCD in assessing isocapnic IH‐induced changes to MCAv. Fifth, given that previous work examining the impact of chronic IH training on EF employed poikilocapnic protocols (Zhang et al. 2025; Wang et al. 2020) compared to the isocapnic protocol used here, it would be interesting for future work to examine whether single bout iso‐ and poikilocapnic protocols differentially impact post‐intervention EF. Last, our EF assessment did not employ an event‐related design to determine whether preparatory phase cortical hemodynamic changes were associated with the observed decrease in antisaccade RTs (see Duschek et al. 2018; Jeyarajan et al. 2024; Tari et al. 2021). Hence, future work could consider measuring post‐IH antisaccade preparatory phase cortical hemodynamics and use the timing of the post‐intervention benefits observed here (i.e., T2) to structure an optimal event‐related design.

## 5 Conclusions

The IH protocol produced a concurrent increase in CBF as estimated via MCAv and CVCi. In turn, between 30 and 45 min following the IH protocol, a reduction in antisaccade—but not prosaccade—RTs was observed and is a result we interpret to reflect a selective EF benefit. These findings indicate that a single bout of IH facilitates EF in healthy young adults, and as such, provides a basis by which future work may examine whether populations at risk for EF deficits due to mobility impairments (e.g., individuals with orthopedic impairment or spinal cord injury) may accrue a similar single bout EF benefit.

## Author Contributions

**Denait Haile:** conceptualization, data curation, methodology, investigation, writing – review and editing, formal analysis. **Nasimi A. Guluzade:** investigation, formal analysis, writing – review and editing. **Antonio Mendes:** investigation. **Daniel A. Keir:** conceptualization, methodology, formal analysis, writing – review and editing. **Matthew Heath:** conceptualization, methodology, software, formal analysis, funding acquisition, supervision, writing – original draft.

## Ethics Statement

This work was approved by the Health Sciences Research Ethics Board, University of Western Ontario (ID: 125185) and was conducted according to the most recent iteration of the Declaration of Helsinki.

## Conflicts of Interest

The authors declare no conflicts of interest.

## Data Availability

The data that support the findings of this study are available from the corresponding author upon reasonable request.

## Endnotes

^1^
In the body of the text, we report only power polynomials attaining a statistical significance of α ≤ .05. For this investigation, only linear effects attained conventional significance, and all other polynomials (e.g., quadratic, cubic etc.) were associated with p values > .092.

^2^
The ability to detect exposure to hypoxia is challenging and is referred to as the “silent killer” in the aerospace industry. As such, military pilots undergo extensive training for the identification of symptoms associated with hypoxic exposure (for review see, Shaw et al. 2021). In support of this, anecdotal evidence from the current study indicated that participants did not detect any somatic changes during the IH protocol and only experienced post‐IH symptomology following the orthostatic change associated with transitioning between the equipment used for the IH protocol and the oculomotor assessment.
